# The relationship between chronic immune response and neurodegenerative damage in long COVID-19

**DOI:** 10.3389/fimmu.2022.1039427

**Published:** 2022-12-16

**Authors:** José Pedro Elizalde-Díaz, Clara Leticia Miranda-Narváez, Juan Carlos Martínez-Lazcano, Eduardo Martínez-Martínez

**Affiliations:** ^1^ Laboratory of Cell Communication & Extracellular Vesicles, Division of Basic Science, Instituto Nacional de Medicina Genómica, Ciudad de México, Mexico; ^2^ Laboratorio de Neurofarmacología Molecular y Nanotecnología, Instituto Nacional de Neurología y Neurocirugía Manuel Velasco Suárez, Ciudad de México, Mexico

**Keywords:** long COVID syndrome, SARS-CoV-2, inflammatory response, neurodegeneration, autoantobodies, autoantigens

## Abstract

In the past two years, the world has faced the pandemic caused by the severe acute respiratory syndrome 2 coronavirus (SARS-CoV-2), which by August of 2022 has infected around 619 million people and caused the death of 6.55 million individuals globally. Although SARS-CoV-2 mainly affects the respiratory tract level, there are several reports, indicating that other organs such as the heart, kidney, pancreas, and brain can also be damaged. A characteristic observed in blood serum samples of patients suffering COVID-19 disease in moderate and severe stages, is a significant increase in proinflammatory cytokines such as interferon-α (IFN-α), interleukin-1β (IL-1β), interleukin-2 (IL-2), interleukin-6 (IL-6) and interleukin-18 (IL-18), as well as the presence of autoantibodies against interferon-α (IFN-α), interferon-λ (IFN-λ), C-C motif chemokine ligand 26 (CCL26), CXC motif chemokine ligand 12 (CXCL12), family with sequence similarity 19 (chemokine (C-C motif)-like) member A4 (FAM19A4), and C-C motif chemokine ligand 1 (CCL1). Interestingly, it has been described that the chronic cytokinemia is related to alterations of blood-brain barrier (BBB) permeability and induction of neurotoxicity. Furthermore, the generation of autoantibodies affects processes such as neurogenesis, neuronal repair, chemotaxis and the optimal microglia function. These observations support the notion that COVID-19 patients who survived the disease present neurological sequelae and neuropsychiatric disorders. The goal of this review is to explore the relationship between inflammatory and humoral immune markers and the major neurological damage manifested in post-COVID-19 patients.

## Introduction

The pandemic caused by the severe acute respiratory syndrome coronavirus 2 (SARS-CoV-2) has increased morbidity and mortality rates worldwide (1, 2). According to various clinical reports and laboratory studies, it is known that the virus can affect different organs such as respiratory tract, lungs, heart, liver, pancreas, kidneys, muscles, and nervous system at different levels (3–5). During the pandemic course, several post COVID-19 effects have been observed that hinder total patient recovery. The World Health Organization (WHO) has denominated these symptoms as long COVID or COVID-19 condition, defining it as a condition that “occurs in individuals with a history of probable or confirmed SARS-CoV-2 infection, usually 3 months from the onset of COVID-19 with symptoms and that last for at least 2 months and cannot be explained by an alternative diagnosis. Symptoms may be new onset following initial recovery from an acute COVID-19 episode or persist from the initial illness. Symptoms may also fluctuate or relapse over time” (6–8).

Several follow-up studies in patients suffering long COVID have documented cardiovascular alterations, fatigue, dyspnea, chest pain, appetite loss and hair loss. Interestingly nervous system seems particularly affected after COVID-19 disease (9, 10). Patients have reported headaches and dizziness, as well as psychiatric disorders and motor discoordination (11–13). In a period of 7 months after viral infection, some patients have presented conditions that are mainly related to neuropsychiatric and neurological deficits, with a prevalence of 19.7% to 36% (4, 14, 15). The characteristic symptoms of these alterations are anosmia, hypogeusia, partial or total hyposmia (16, 17), myalgia, cerebral inflammation, cerebrovascular strokes (18), acute encephalopathy, seizures, Guillain-Barré syndrome (19), neurocognitive disorders, sleep disorders, delirium, memory deficit, concentration deficit, depression, psychosis, hallucinations, paranoia (20), chronic fatigue and partial or total apraxia (21).

Similar to the neurological alterations of SARS-CoV-2 post-infection, there are data from patients who were infected with SARS-CoV-1 and MERS. The clinical follow-up carried out on these patients recorded symptoms of depression, disorder of post-traumatic stress (PTSD), anxiety, sleep disorders, weakness, chronic fatigue and general pain, in a follow-up period covering 6 to 20 months post-infection (22, 23), symptoms set similar to the neurological alterations reported in SARS-CoV-2 post-infection. A meta-analysis of 120,970 patients infected with SARS-CoV-2 revelated that women are more susceptible to present moderate neurological and cardiovascular long-COVID symptoms. It also was reported that age is directly related to a higher incidence of psychiatric, respiratory, digestive and skin conditions. In addition, in a subgroup of 106,284 participants it was observed an incidence of 19.7% of neurological disorders, where the main manifestations included, concentration difficulty (14.6%), headache, disorders of the taste and smell, cognitive impairment, memory deficits, dizziness, and cramps. Furthermore, psychiatric conditions affected 20.3% of the participants, who presented PTSD, depression, sleep disorder and anxiety (14).

The analysis of cerebrospinal fluid (CSF) and peripheral blood samples of 127 patients, who were positive for SARS-CoV-2 and showed neurological damage symptoms after 7 days of infection, revelated that they suffered systemic inflammation and impaired blood-brain barrier (BBB). The neurological manifestations included encephalopathy, altered consciousness, delayed walking reaction, epilepsy-like electroencephalogram (EEG) changes, cerebral ischemia, myelitis, cerebellar ataxia, sensorimotor symptoms of unknown cause, cognitive impairment, peripheral neuropathy, anosmia, headache and nausea (24). Altogether these studies indicate a relationship between SARS-CoV-2 infection and neurological conditions observed in long COVID. The main goal of this review is to elucidate the role of the antiviral dysregulation response by the immune system and its relationship with the sequelae of damage to the central nervous system (CNS) in patients with long COVID.

## Relationship between SARS-CoV-2 and nervous system

It has been documented that coronaviruses have the ability to affect the CNS (25). In this context, several investigations have discovered that β-coronaviruses such as MERS-CoV and SARS-CoV-1 can infect the CNS (25–29). Furthermore, traces of SARS-CoV-2 have been detected in the olfactory mucosa, trans olfactory mucosa, neuronal projections and neurons during and after the infection period (30–34). In some COVID-19 cases the first symptoms presented by patients is hyposmia or anosmia. This could be due to the olfactory epithelium damage caused by the coronavirus, which in turn affects the olfactory neural network that is connected with the primary olfactory cortex (35–37). To date there is no precise understanding about the dynamics of the initial antiviral response against SARS-CoV-2 that occur at the level of the olfactory epithelium. However, there are data from nasal samples that showed an increase of proinflammatory cytokines within two days after the first symptoms, compared with samples of same tissue that were taken at longer times (5 or more days after presenting the first symptoms), when the levels of proinflammatory cytokines decreased (17). This could indicate that the immune response produced in the olfactory epithelium associated with nerve cells occurs in a transient manner. However, this response is sufficient to generate some neuronal damage either by a direct action of the virus or by an indirect mechanism that involves the dysregulation of the immune response.

The BBB is the main physiological structural interconnection between the external environment and the brain whose main function is to protect central neurons. It also participates in the selective transit of cells, nutrients and brain cell metabolism toxic byproducts (38). When a systemic inflammation process occurs, the BBB induces a series of brain responses whose main objective is to promote brain survival, which is known as disease behavior (39). This response induces a set of physiological and behavioral changes, coordinated and executed by the brain, which protect the individual from the various phases occurring during an infection. For example, the induction of lethargy allows to fight infection through the induction of fever and anorexia (40, 41).

In patients who succumbed to COVID-19 and who had an exacerbated inflammatory response, presented BBB involvement manifested through multifocal vascular damage caused by autoantibodies. This process that induced serum proteins infiltration into the brain parenchyma, generalized endothelial cell activation, classical complement pathway activation, platelet aggregates and microthrombi adhered to endothelial cells throughout the vascular lumen. In addition, the infiltration of macrophages, T cells and B cells into brain structures has been reported, observing a greater presence of CD8+ T cells in the perivascular region compared to CD4+ cells. There are also reports of astrogliosis in perivascular regions and microglial nodule formation in the hindbrain, which is associated with focal neuronal loss and neuronophagia (42).

The SARS-CoV-2 induces a nuclear structure reorganization and the dispersion of the genomic compartments of the cell, which leads to the low expression of the genes *ADCY3*, *CNGA2*, *GN13*, *GFY*, *OMP*, *LHX2* and *ATF5*, which are key in the olfactory receptors signaling and this downregulation lead to anosmia (17). It has been proposed that once the virus enters the olfactory receptor neurons, the infection is propagated through the synaptic connections (43). In the case of the olfactory receptor neurons-mitral cells axis, there is an activation of the glial, which in turn promote the release proinflammatory cytokines such as IFN-α, TNF-α, IL-1α, IL-1β, IL-2, IL-6, IL-8, IL-17A, IL-18, CXCL10, CXCL12, CCL1, CCL2, CCL3, CCL4, CCL5, CCL7, CCL11, GM-CSF and B cell-activating factor belonging to the TNF family (BAFF). These cytokines that have been detected at elevated levels in samples of CSF, brain tissue, and serum of peripheral blood from patients with severe COVID-19 (44–49). It should be noted that the upregulated production of these cytokines can cause serious damage to the CNS, since it promotes neuronal stress and apoptosis, as well as the interruption of the BBB (43). In a mild respiratory COVID mouse model, it was observed that these events eventually increase neuroinflammation cascades causing synaptic loss, demyelination, excitotoxicity and transcriptional downregulation of *Trem2*, *Sall3* and *Adrb1* genes in microglia, the latter gene being an indicator of white matter degeneration (48). Other cerebral regions can potentially be affected by a similar mechanism. For instance, midbrain dopamine neurons derived from human pluripotent stem cells are selectively permissive to SARS-CoV-2 infection. This triggers an inflammatory response at neuronal level and the expression of the insulin like growth factor binding protein 7 (IGFBP7) and LAMININ B1 genes associated with cellular senescence (32). The expression of these molecules leads to the overactivation of glia and trigger mechanisms of neuronal damage (50). Overall, the neuronal damage associated with the upregulation of proinflammatory cytokines could be the cause of the appearance of neurological symptoms related with long COVID (**Table 1**).

**Table 1:** Upregulated cytokines associated at neurological damage observed in patients with long COVID.

Neurological affectation	Upregulated cytokines	References
Neurocognitive disorders	IFN-α, IL-1, IL-6, IL-17A, IL-18, CCL7	(51–59)
Sleep disorders	IL-1, IL-8, IL-18	(55, 56, 58, 60)
Memory deficit	IL-1, IL-18, CCL3, CCL7, BAFF	(54, 57, 60–63)
Concentration deficit	IFN-α, CCL7	(51, 57, 64)
Depression	IFN-α, TNF-α, IL-1, IL-2, IL-6, IL-8, IL-17A, IL-18, CCL1, CCL2, CCL5, CCL7, CCL11	(51, 52, 54–57, 65–71)
Psychosis	IFN-α, IL-6, BAFF	(51, 55, 63, 72)
Hallucinations	IFN-α	(51)
Systemic inflammation	IFN-α, TNF-α, IL-1, IL-2, IL-6, IL-8, IL-12, IL-17A, IL-18, CXCL10, CCL3, CCL4, CCL5, CCL7, GM-CSF	(51, 54–56, 60, 61, 73–78)
Peripherial neuropathy	TNF-α, IL-1, IL-2, IL-6, IL-8, IL-12, IL-17A, IL-18, CXCL10, CCL3, CCL4, CCL5, CCL7, GM-CSF	(54–56, 60, 61, 73–79)
Stroke	IFN-α, TNF-α, IL-1, IL-6, IL-8, IL-17A, IL-18, CXCL-10, CXCL12, CCL2, CCL3, CCL5, CCL11	(55, 56, 60, 75, 78, 80–82)
Anxiety	TNF-α, IL-1, CXCL12	(56, 67, 83)

The effects that SARS-CoV-2 infection induces in brain structures was analyzed on 401 patients who suffered from COVID-19. Using the UK Biobank database, there was a selection of patients with brain imaging studies prior to COVID infection, and all patients were subject to brain imaging 38 months later. All the patients had at least one or more of the following affectations: significant reduction in gray matter thickness and tissue contrast in the orbitofrontal cortex, changes in diffusion measures, which are indicators of tissue damage, increase in CSF volume and overall size brain reduction (37). These changes were consistent and related to previously detected cognitive impairment in the study population. SARS-CoV-2 infection also changes the vasculature of the brain, since one of the damages induced by the virus is ischemic and hemorrhagic cerebrovascular strokes (84). A postmortem study in patients who died from severe COVID-19 revelated the presence of viral inclusion structures, accumulation of inflammatory cells in the vascular endothelium (lymphocytic endotheliitis), and endothelial cell apoptosis (50). All these sequelae of SARS-CoV-2 infection in the CNS has been monitored in the serum and CSF of patients with long COVID who present neurological damage symptoms (encephalopathy, seizures, paraplegia, paresis, Guillain-Barré syndrome, ataxia and dysesthesia). These patients show a slight increase in white blood cells and an increase in the concentrations of total proteins and albumin, which indicates that the virus triggers a systemic dysfunction that can be detected at blood and CSF level (24).

## Deciphering the process of neurological damage caused by the exacerbated innate immune response to SARS-CoV-2

Once a virus reaches the nerves and brain tissue, an inflammatory mechanism is activated which aims to limit the infection process, eliminate the virus, or repair cell damage. Depending on the activated immunological pathway and the magnitude with which it is activated, the response can have positive or negative consequences on the physiology and behavior of the individual (85). The complications of exacerbated neuroinflammation can include headache, ischemia, interstitial edema, cerebral vasodilatation, blood vessel injury, vomiting, visual loss, blood stasis, increased cerebral pressure, cognitive problems, and loss of consciousness (86–89). Neuroinflammation characterized by an early and brief inflammatory response is considered neuroprotective, and is initiated by the activation of glial and endothelial cells (90, 91). On the contrary, a prolonged neuroinflammatory activation induces damage to brain structures and tissues, which has been associated with several neurodegenerative diseases, such as Alzheimer’s disease (AD), Parkinson’s disease (PD), and multiple sclerosis (92, 93).

The role of the microglia during resting conditions is to constantly examine the brain microenvironment to maintain homeostasis through the elimination of cellular waste (94). When there is a damage to neuronal structures, a process known as microglia activation occurs. This process is characterized by the release of cytokines, chemokines, and inflammatory molecules (95). However, when the immune response is dysregulated, the exacerbated release of proinflammatory cytokines occurs, which has been associated with high mortality in patients with COVID-19 (96). This type of patients show microglia hyperactivation through multisystem inflammatory syndrome (97, 98) and systemic inflammatory response syndrome (99).

Dysregulation of the immune response due to the SARS-CoV-2 infection has the ability to downregulate angiotensin converting enzyme 2 (ACE-2) expression, which influences the activation and balance of the inflammatory pathway (100). The decreased expression of ACE-2 increases the concentration of Ang-II favoring the ACE/Ang-II/AT1R pathway. This leads to the activation of the NF-κB transcription factor and the consequent activation of the production and release of proinflammatory cytokines (101). Altered cytokine concentrations have been observed in samples of both patients with acute SARS-CoV-2 and in patients with manifestations associated with long COVID (43, 102, 103). The increase in Ang-II concentration also favors the Ang-II/aminopeptidase-A/Ang-III/aminopeptidase-A/Ang-IV/AT4R pathway (104, 105). The increase in Ang-III concentration induces hormone overproduction such as vasopressin in the hypothalamus and aldosterone in the adrenal gland (105). These alterations result in increased peripheral vascular resistance and blood pressure. Moreover, Ang-III dysregulates Na+/K+ equilibrium which results in vascular damage, stroke and heart attack (106, 107). Both Ang-III and Ang-IV can bind to AT1R, thus induce the activation of this receptor and the activation consequently of the NF-κB transcription factor (105, 108, 109). The increase of Ang-IV dysregulates the vasodilatation process, increases the excretion of sodium, and the release of plasminogen activator inhibitor-1, favoring the development of thrombotic events both in lungs and in the brain (108, 110–113). According to transcriptome databases, ACE2 is expressed in excitatory and inhibitory neurons, astrocytes, oligodendrocytes, and endothelial cells (114). We believe that ACE-2 downregulation induced by SARS-CoV-2 infection, is one of the first pathways responsible for immunological response damage to the CNS.

An additional mechanism associated with pro-inflammatory cytokines induction occurs when the virus infects the cell, and the innate immune system detects viral RNA genome, either as ssRNA or one of dsRNA’s intermediaries through the Toll-Like Receptors including TLR3, TLR7, and TLR8 (115, 116). These receptors are responsible for activation of transcription factors such as IRF3, IRF7, NF-κB, ISRE3, and API. This transcription factors are related to the expression of key proinflammatory cytokines in the antiviral response such as TNF-α, IFN-α, IFN-β and IFN-γ (115, 117). IFN-α and IFN-β activates genes involved in apoptosis processes, in the modulation of immune response, in cellular attraction and adhesion, and genes involved in antiviral and pathogenic detection (118). The balance that exists between IFN-α and IFN-β concentrations is key in the regulation of the inflammatory response. If there is any imbalance in their concentrations, the IFN-γ production is affected and therefore the anti-inflammatory process does not occur. In addition a chronic inflammation is promoted when the humoral response is deficient (119). Interestingly, in samples of respiratory epithelial cells and plasmacytoid dendritic cells from patients with severe COVID-19, there is a decrease of type-I IFNs associated with self-recessive deficiencies in genes that code for the proteins involved in interferon production (e.g. TLR3, UNC93B, TRIF, TBK1, TBK1, IRF3, IRF7, IFNAR1/2, MYD88, GATA2 and IRAK4) (120–126). In CSF samples of patients with acute COVID-19 and sings of neurological damage, it was found a reduced interferon response, expansion of clonal T cells and a depletion of CD4+ T cells (127). Thus, it is possible that the interferon production during and after infection is a key point in the process of regulating systemic and neuronal inflammation.

The inflammatory response in the CNS system is mediated by resident microglia and astrocytes (128), which detects the presence of an exogenous or pathogenic agent such as SARS-CoV-2 (129). Besides its direct participation in the elimination of an infection, the microglia establish the balance between the innate immune response and the adaptive immune response (130, 131). During acute COVID-19, the exacerbated release of proinflammatory cytokines promotes the production of reactive oxygen species (ROS), which causes stress and cell damage at the systemic level, affecting brain tissue (129). In some COVID-19 patients these cellular events manifest in symptoms such as ischemia, inflammation of brain tissue, obstruction of blood flow, headaches, loss of consciousness, cerebral edema, and neuronal death (131–133).

Previous studies have reported that during influenza virus infection there is an increase in the levels of proinflammatory cytokines, such as IL-1β, IL-6, CXCL8, CXCL9, CXCL10, CCL2, and TNF-α, in the CSF of patients who present neurological alterations such as acute encephalitis and encephalopathy (134, 135). It is also known that patients infected with human orthopneumovirus and presenting neurological symptoms such as encephalitis and encephalopathies, have elevated levels of the proinflammatory cytokines IL-6, IL-8, CCL2, and CCL4 in CSF samples (136, 137). West Nile virus is also known to cause a neuroinvasive disease manifesting meningitis, meningoencephalitis, encephalitis, or acute flaccid paralysis, commonly associated with diarrhea/vomiting, weakness, impaired vision, confusion, or drowsiness, and shows elevated levels of proinflammatory cytokines IL4, IL6, and IL10 in serum samples (138). Finally, Zika virus can infect the CNS and induce microcephaly in fetuses and rare but serious neurological diseases in adults, which are associated with excessive production of IFN-α, IFN-β, IL-6, and TNF-α (139).

Interestingly, these neuroinflammatory pathological processes observed in long COVID patients, resemble those that occur in early phase of Parkinson’s disease (PD and AD (92). For example, high levels of TNF-α and low levels of TNF-β have been detected in CSF samples from patients with mild cognitive impairment who progressed to AD, and the cytokines IL-1β, IL-6, and TNF-α, tend to increase slowly, while the cytokines IL-18, MCP-1, and IP-10 peak at a certain stage of the disease (140, 141). Activation of microglial cells has been detected in the substantia nigra of patients with PD, due to the fact that aggregated α-synuclein is released from the damaged dopaminergic neurons (142). The accumulation of α-synuclein leads microglia to a reactive proinflammatory phenotype in which TNF-α, nitric oxide, and IL-1β are produced, generating a neuroinflammatory state as recently shown in an *in vitro* model of PD (143).

## Role of the dysregulated antibodies response against SARS-CoV-2 infection in neurological disorders

Part of neurological sequelae previously mentioned suffered by SARS-CoV-2 patients, were also reported in individuals who survived SARS-CoV-1 infection in 2004 who presented cerebrovascular disorders such as ischemic stroke (144). These affectations could be caused by abnormalities in coagulation and hyperinflammation promoted by the presence of antiphospholipid autoantibodies (eg. antiphosphatidylserine or antiprothrombin) produced by plasma cells (88, 145, 146). Autoantibodies are a type of antibodies that recognize epitopes present in organs or tissues of the same individual and are related to the development of autoimmune diseases including allergies and oncopathologies (147, 148). Much of the generation of these autoantibodies is caused by genetic mutations, infections or environmental factors (149). The autoantibody generation can result from an altered production of cytokines, stimulation of toll-like receptors, or pattern recognition receptors (150). Furthermore, they can also originate from an inadequate and dysregulated release of autoantigens by cells and tissues, and/or molecular mimicry (150, 151). In the case of COVID-19 infection, various studies indicate that the spike protein of SARS-CoV-2 is the causal agent of inducing the autoantibodies generation, which might be a common characteristic in coronavirus infections (147, 148, 150, 152). It has been reported that the antibodies produced by plasma cells against spike protein or receptor-binding domain of the SARS-CoV-2 can cross-bind with own antigens (153). In a follow-up study of 610 patients after 6 to 12 months post-infection with SARS-CoV-2, there were low concentrations of IgM and IgG3 that correlated with a predisposition to develop long COVID. Moreover, 71% of these patients presented severe COVID-19 and bronchial asthma at the same time (152). Regarding these immunoglobulins, it is known that both are induced by the controlled production of interferons and antagonized by IL-14 (154, 155). In addition, IgMs have a relevant role in the humoral response since it is the first immunoglobulin that participates in pathogen elimination (156). IgMs functions as a powerful complement activator, participate in the activation and regulation of the inflammatory response, opsonization, and destruction of pathogens present in the circulatory system (155, 157). In addition, IgMs are associated with the protective mechanisms of the vasculature and mucous membranes (157). IgG3s, activate the complement system and have a great affinity with Fc receptors (158). The deficiency of IgG3s is related with the development of autoimmune diseases (159). This could indicate that the innate immune response dysregulation directly affects the humoral response activation process, which leads to a deficient, non-specific and delayed production of antibodies against SARS-CoV-2.

In a recent multicenter study it was proposed that a deficient and prolonged immune response in hospitalized severe COVID-19 patients promotes the adaptive immune response that attacks non-structural viral proteins and causes the development of IgG autoantibodies (160). Similarly, a proteomic profiling analysis revealed that the generation of certain autoantibodies (e.g. MUC1 or TNFRSF6B) is associated with the severity of the disease (147). Consistent with this notion, several investigations have also found that patients who had COVID-19 exhibit marked increases in autoantibody reactivity compared with uninfected individuals (160, 161). These individuals show a high prevalence of autoantibodies against immunomodulatory proteins (including cytokines, chemokines, complement components, and cell surface proteins) (162). The main consequence of these autoantibodies is the disruption of the immune function and the impairment of the virologic control by inhibiting immunoreceptor signaling and altering the composition of peripheral immune cells (163, 164).

There are cases where the presence of autoantibodies can be detected prior to any viral infection, suggesting a genetic predisposition to the generation of these autoantibodies (165). This could explain why some COVID-19 patients are more susceptible to produce autoantibodies that promote long COVID (166, 167). Recent studies have shown that some of these autoantibodies have an affinity for blood vessel and nervous system proteins, which could explain the neurological effects of long COVID by two mechanisms (168). First, autoantibodies could potentiate the cellular stress induced by proinflammatory cytokines. Second, autoantibodies could cause specific and long-term damage in patients suffering from post-COVID neurological sequelae (43, 168). In fact, COVID-19 patients with neurological sequelae produce autoantibodies that inhibit the function of key proteins involved in neuroprotection processes, neurite outgrowth, axogenesis, neuronal plasticity, neurotransmission, neuronal survival, and axonal regeneration (**Supplementary Table 1**) (167). The generation of these autoantibodies may aggravate the neuronal damage.

The dysregulation of the immune response and the deficient elimination of cells infected by SARS-CoV-2 promote the release of autoantigens towards the extracellular space and the consequent generation of autoantibodies (169, 170). The analysis of the “autoantigenicoma” in patients who suffered from COVID-19 through the detection of autoantigens bound to determatan sulfate (autoantigen-DS complex) seems to be helpful to predict the appearance of autoimmune diseases and neurological damage (171, 172). Using this strategy, 751 autoantigen candidates were found, of which 657 are directly altered by infection with SARS-CoV-2. Remarkably, 400 of those autoantigens are related to autoimmune diseases and cancer (162). Regarding the nervous system, 150 autoantigens of proteins are related to axon guidance, neuron projection, myelin sheath, axon growth cone, neuronal cell body, cerebellar Purkinje cell layer, peripheral nervous system axon regeneration, radial glial scaffolds and proteins related to the olfactory bulb. There were also 193 autoantigens of proteins related to neurological diseases such as neuronal infection with Japanese encephalitis virus, neuroblastoma, glioblastoma, neurodegeneration in Down syndrome, AD, schizophrenia, cerebral ischemia induced neurodegenerative diseases, PD, and neurodegeneration (**Supplementary Table 2**) (172). The mechanism by which coronaviruses could resemble conditions of early events of neurodegeneration should be explored considering the participation of the immune system and the uncontrolled generation of autoantibodies that deteriorate neuronal circuits.

## Summary and proposal

**Figure 1:**
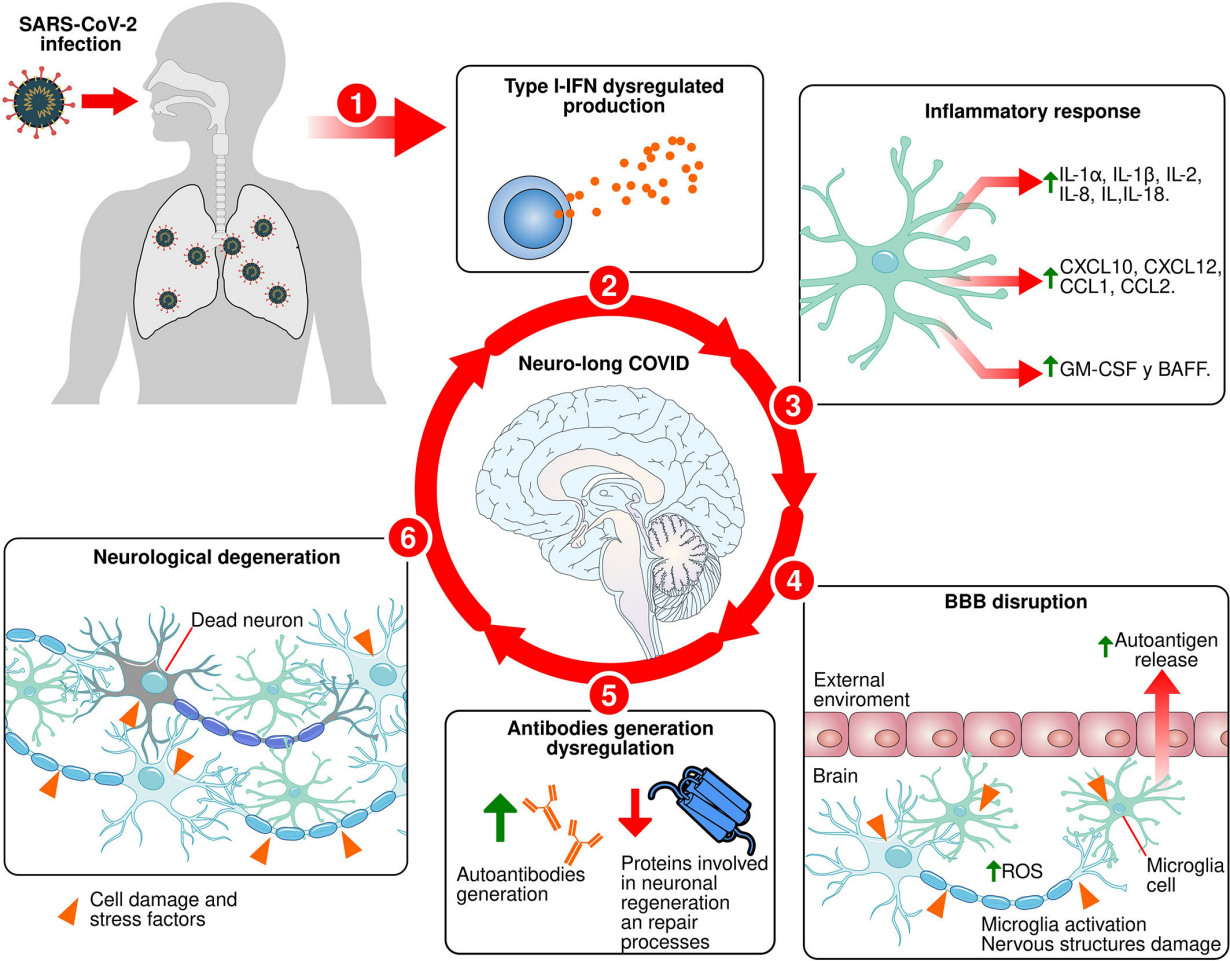
Proposed mechanism for neuro-long COVID. 1: SARS-CoV-2 infects olfactory epithelial and lungs. 2: Type-I IFNs production dysregulated during primary immune response process against SARS-CoV-2 infection. 3: Exacerbated release of proinflammatory cytokines. 4: The exacerbated and dysregulated inflammatory response causes the proinflammatory molecules release that damage the BBB, facilitate the infiltration of immune cells into brain tissue, activate microglia, and damaging brain tissue cells, causing the autoantigens release. 5: Innate immune response dysregulation affects the humoral response activation process and induce a nonspecific and delayed production of antibodies against SARS-CoV-2 and the generation of autoantibodies against key proteins involved in neuronal regeneration and repair processes. 6: Induction of neuronal death in specific areas.

The effects of long COVID on the CNS are increasingly evident. For this reason, in the present work we analyzed the role of the immune response against the coronavirus and its impact on neuronal structures. The SARS-CoV-2 infects olfactory epithelial cells through ACE-2 (173). Through genetic rearrangements, the virus downregulates the expression of proteins such as olfactory receptors and ACE-2 (17, 100). The latter is implicated in the production of proinflammatory cytokines (43). When the immune system detects the entry of the virus, it activates the primary response, which is characterized by the release of proinflammatory cytokines and the activation of immune cells. These processes are regulated by type-I INFs and together with IFN-γ (115, 117) induce the generation of antibodies (130, 131). However, due to the downregulation of ACE-2 and mutations in type I INFs, the inflammatory response is dysregulated, provoking the exacerbated release of proinflammatory cytokines (117). This response damages cellular structures and promotes the release of autoantigens (168, 169). At the same time, the dysregulation of the innate immune response affects the activation process of the humoral response (119, 169). This may lead to a nonspecific and delayed production of antibodies against SARS-CoV-2 and the generation of autoantibodies that recognize key proteins involved in neuronal regeneration and repair processes, thereby increasing neurodegeneration (167). We think this generates a cyclical process of recognition and destruction of neuronal structures (**Figure 1**). Depending on the region that is affected, this promotes the appearance of neurological symptoms observed in patients with long COVID.

## Author contributions

JM-L and CM-N wrote the manuscript. JE-D and EM-M conceived and wrote the manuscript. All authors contributed to the article and approved the submitted version.
