# Supplementary material for: The relationship between chronic immune response and neurodegenerative damage in long COVID-19
Source: Front Immunol. 2022 Dec 16;13:1039427. doi: 10.3389/fimmu.2022.1039427 (PMC9800881; doi:10.3389/fimmu.2022.1039427)
Supplement: Supplementary file 1 [file Table_1.docx]

**Supplementary tables**

Supplementary Table 1: Key proteins that are target of autoantibodies generated from SARS-CoV-2 infection

| **Protein** | **Normal function in the nervous system** | **Reference** |
| --- | --- | --- |
| Pituitary adenylate cyclase-activating polypeptide  (PACAP) | Regulates neuronal physiology through Gs/Gq-coupled receptors. Participates in affective processing, neuroprotection, and cognition, in hypothalamic, limbic, and mnemonic systems. | (1, 2) |
| Amyloid precursor protein  (APP) | Cell surface receptor involved in cell mobility, transcription regulation, axonal transport, copper homeostasis, oxidative stress regulation, neurite growth, neuronal adhesion and axonogenesis. | (2, 3) |
| Carboxypeptidase E  (CPE) | Cleaves C-terminal amino acid residues and is involved in the biosynthesis of neurotransmitters. It also act as a neurotrophic factor in the neuroprotection and neuronal survival. | (2, 4) |
| G protein-coupled receptor 6  (GPR6) | Promotes axonal regeneration, neurite outgrowth and blocks myelin inhibition in neurons. | (2, 5) |
| Metabotropic glutamate receptor 5  (mGluR5) | The receptor activates a phosphatidylinositol-Ca second messenger system. Furthermore is involved in the regulation of neural network activity and synaptic plasticity. | (2, 6) |
| Orexin receptor type 2  (Ox2R) | Binds to the hypothalamic neuropeptides orexin A and orexin B, triggering an increase in cytoplasmic Ca(2+) levels. Involved in feeding behavior regulation. | (2, 7) |
| Cell Adhesion Molecule 3 (SynCAM3) | Regulates axon guidance, myelination, and maintenance of the axonal architecture. Intercellular contact between Schwann cells and their underlying axons is mediated by binding of glial SynCAM4 to axonal SynCAM3 in the peripheral nervous system. | (2, 8) |
| Kin of IRRE-like protein 3 | Adhesion molecule required for formation of target-specific synapses at hippocampal mossy fiber filopodia, the synaptic structures connecting dentate granule and GABA neurons. | (2, 9) |
| Lymphocyte antigen 6 family member H  (Ly-6H) | Could regulate the nicotinic acetylcholine receptors (nAChR) activity, since in vitro it inhibits the maximum response of the nAChRs that contain alpha-3:beta-4, furthermore to participating in the intracellular trafficking of nAChRs that contain alpha-7 by inhibiting its expression on the cell surface, thereby inhibiting alpha-7/CHRNA7 signaling in hippocampal neurons. | (2, 10) |
| Neuronal growth regulator 1 | Participates in cell-adhesion, in addition to acting as a trans-neural growth-promoter in regenerative axons sprouting in the mammalian brain. | (2, 11) |
| Neuropilin and tolloid like 1 | Participates in the development and maintenance of neural circuits. In the spatial learning and memory context, regulates long-term NMDA receptor-dependent synaptic plasticity and cognition. | (2, 12) |
| Neurexophilin-1 | Neuropeptide-like signaling molecules and bind to alpha-neurexins and possibly other neuroreceptors. | (2, 13) |
| Mu-type opioid receptor  (MOR-1) | Endogenous opioids receptor such as beta-endorphin and endomorphine. Mediates inhibition of voltage-gated calcium channels in the central presynaptic terminals of primary afferent nociceptors, mechanism that mediate analgesia at the spinal level. | (2, 14) |
| Oxytocin-neurophysin 1  (OT-NPI) | Neuropeptide precursor that is processed to oxytocin and neurophysin I. OXT is expressed in several areas of the CNS. Promotes the interaction, complex social cognition and the social recognition, addition to exercising anxiolytic and antidepressant effects. Also influences hippocampal synaptic plasticity, circadian rhythms, autonomic responses, antinociception, and motor neuron excitability. | (2, 15) |
| Neuroendocrine convertase 1  (NEC 1) | The enzyme has an overriding participation in the proteolytic activation of polypeptide hormones and neuropeptide precursors. | (2, 16) |
| Pro-melanin concentrating hormone  (Pro-MCH) | Preproprotein that is proteolytically processed to generate multiple products, like melanin-concentrating hormone, neuropeptide-glutamic acid-isoleucine, and neuropeptide-glycine-glutamic acid, wich may act as a neurotransmitters or neuromodulators in several neuronal regulation functions such as food intake, and general arousal. | (2, 17, 18) |
| Pleiotrophin  (PTN) | Regulates many processes like the oligodendrocyte precursor cell differentiation through PTPRZ1 binding, enhancing the phosphorylation of AFAP1L2 that activate the PI3K-AKT pathway, promotes dendritic arborization in adult hippocampus, spine development, and functional integration and connectivity of newborn granule neurons through activating AKT signaling pathway. Binds GPC2 and chondroitin sulfate proteoglycans at the neuron surface, leading to abrogation of binding between PTPRS and CSPGs and neurite outgrowth promotion. Furthermore, inhibits proliferation and enhances differentiation of neural stem cells by inhibiting FGF2-induced fibroblast growth factor receptor signaling pathway. | (2, 19) |
| Reticulon-4 receptor  (NgR) | Regulates axon regeneration and neuronal plasticity in the adult central nervous system, participates in postnatal brain development, the normal axon migration across the brain midline and normal formation of the corpus callosum, protects motoneurons against apoptosis, participates in regulating neuronal precursor cell motility during cortical development, furthermore to restricts the number dendritic spines and the number of synapses that are formed during brain development. | (2, 20) |
| Reticulon-4 receptor-like 1  (NgR3) | Has a important role in postnatal brain development and in regulating axon regeneration in the adult central nervous system. Participates in normal axon migration across the brain midline and normal formation of the corpus callosum. Protects motoneurons against apoptosis. Plays a role in inhibiting neurite outgrowth and axon regeneration via its binding to neuronal chondroitin sulfate proteoglycans. Restricts the number dendritic spines and the number of synapses that are formed during brain development. | (2, 20) |
| Prokineticin-1 | Induces proliferation and differentiation of enteric neural crest cells. Promotes the proliferation and migration of neuroblastoma cells. Positively regulates PTGS2 expression and prostaglandin synthesis. | (2, 21) |
| Glia-derived nexin  (GDN) | Regulates the migration of neuronal cells and promotes neurite extension. | (2, 22) |
| Neuroserpin | Has a neuroprotective role in perinatal hipoxia-ischaemia and adult stroke. Participates in physiological human brain development, since involved in the formation or reorganization of synaptic connections as well as for synaptic plasticity. | (2, 23) |

Supplementary Table 2: Autoantigens of proteins related to neurological diseases, detected in patients with COVID-19

| **Protein** | **Nervous system related disease** | **Reference** |
| --- | --- | --- |
| Actin, cytoplasmic 1 | Glioblastoma, neurodegeneration, neurodegenerative cerebral ischemia | (24) |
| Annexin A2 | Schizophrenia, neurodegeneration |  |
| Calreticulin  (CRP55) | Neurodegenerative cerebral ischemia, glioblastoma |  |
| Cathepsin B  (APPS) | Glioblastoma, neuroblastoma, neurodegeneration |  |
| Cathepsin D | Glioblastoma, neuroblastoma, neurodegeneration |  |
| Elongation factor 1-alpha 1 | Alzheimer's disease |  |
| Elongation factor 2  (EF-2) | Neurodegeneration |  |
| Glyceraldehyde-3-phosphate dehydrogenase  (GAPDH) | Viral encephalitis, schizophrenia, Parkinson's disease |  |
| HSPA1B Heat shock 70 kDa protein 1A | Glioblastoma, Alzheimer's disease |  |
| Endoplasmic reticulum chaperone BiP  (GRP-78) | Viral encephalitis, glioblastoma, neurodegeneration, neuroblastoma, neurodegenerative cerebral ischemia |  |
| Heat shock protein beta-1  (HspB1) | Neurodegeneration, neurodegeneration |  |
| Heat shock protein HSP 90-alpha  (HSP86) | Alzheimer's disease, glioblastoma, viral encephalitis, neurodegenerative cerebral ischemia |  |
| Integrin beta-1  (GPIIA) | Glioblastoma |  |
| Prelamin-A/C | Neurodegeneration, Alzheimer's disease |  |
| Microtubule-associated protein 1B  (MAP-1B) | Alzheimer's disease |  |
| Myosin-9 | Glioblastoma |  |
| Nucleosome assembly protein 1-like-1 | Neuroblastoma |  |
| Nucleophosmin  (NPM) | Viral encephalitis |  |
| Poly(A)-biding protein 1  (PABP-1) | Parkinson's disease |  |
| Protein disulfide-isomerase A3 | Viral encephalitis, neurodegeneration |  |
| Protein disulfide-isomerase A4 | Viral encephalitis |  |
| Profilin-1 | Neurodegeneration |  |
| Plectin-1  (PLTN) | Alzheimer's disease |  |
| Protein disulfide-isomerase  (PDI) | Viral encephalitis |  |
| Vimentin | Neurodegenerative cerebral ischemia, schizophrenia |  |

**References**

1. Gilmartin MR, Ferrara NC. Pituitary Adenylate Cyclase-Activating Polypeptide in Learning and Memory. Frontiers in Cellular Neuroscience. 2021;15.

2. Wang EY, Mao T, Klein J, Dai Y, Huck JD, Jaycox JR, et al. Diverse functional autoantibodies in patients with COVID-19. Nature. 2021;595(7866):283-8.

3. Baumkötter F, Schmidt N, Vargas C, Schilling S, Weber R, Wagner K, et al. Amyloid Precursor Protein Dimerization and Synaptogenic Function Depend on Copper Binding to the Growth Factor-Like Domain. The Journal of Neuroscience. 2014;34(33):11159.

4. Sharma VK, Yang X, Kim S-K, Mafi A, Saiz-Sanchez D, Villanueva-Anguita P, et al. Novel interaction between neurotrophic factor-α1/carboxypeptidase E and serotonin receptor, 5-HTR1E, protects human neurons against oxidative/neuroexcitotoxic stress via β-arrestin/ERK signaling. Cellular and Molecular Life Sciences. 2022;79(1):1-19.

5. Tanaka S, Ishii K, Kasai K, Yoon SO, Saeki Y. Neural expression of G protein-coupled receptors GPR3, GPR6, and GPR12 up-regulates cyclic AMP levels and promotes neurite outgrowth. Journal of Biological Chemistry. 2007;282(14):10506-15.

6. D’Antoni S, Spatuzza M, Bonaccorso CM, Musumeci SA, Ciranna L, Nicoletti F, et al. Dysregulation of group-I metabotropic glutamate (mGlu) receptor mediated signalling in disorders associated with Intellectual Disability and Autism. Neuroscience & Biobehavioral Reviews. 2014;46:228-41.

7. Sakurai T, Amemiya A, Ishii M, Matsuzaki I, Chemelli RM, Tanaka H, et al. Orexins and orexin receptors: a family of hypothalamic neuropeptides and G protein-coupled receptors that regulate feeding behavior. Cell. 1998;92(4):573-85.

8. Sukhanov N, Vainshtein A, Eshed-Eisenbach Y, Peles E. Differential Contribution of Cadm1–Cadm3 Cell Adhesion Molecules to Peripheral Myelinated Axons. The Journal of Neuroscience. 2021;41(7):1393.

9. Martin EA, Muralidhar S, Wang Z, Cervantes DC, Basu R, Taylor MR, et al. The intellectual disability gene Kirrel3 regulates target-specific mossy fiber synapse development in the hippocampus. eLife [Internet]. 2015 2022/08/12; 4:[e09395 p.]. Available from: https://doi.org/10.7554/eLife.09395.

10. Horie M, Okutomi K, Taniguchi Y, Ohbuchi Y, Suzuki M, Takahashi E-i. Isolation and Characterization of a New Member of the HumanLy6Gene Family(LY6H). Genomics. 1998;53(3):365-8.

11. Cheon Y, Yoo A, Seo H, Yun S-Y, Lee H, Lim H, et al. Na/K-ATPase beta1-subunit associates with neuronal growth regulator 1 (NEGR1) to participate in intercellular interactions. BMB Rep. 2021;54(3):164-9.

12. Ng D, Pitcher GM, Szilard RK, Sertié A, Kanisek M, Clapcote SJ, et al. Neto1 Is a Novel CUB-Domain NMDA Receptor–Interacting Protein Required for Synaptic Plasticity and Learning. PLOS Biology. 2009;7(2):e1000041.

13. Petrenko AG, Ullrich B, Missler M, Krasnoperov V, Rosahl TW, Südhof TC. Structure and evolution of neurexophilin. J Neurosci. 1996;16(14):4360-9.

14. Mestek A, Hurley JH, Bye LS, Campbell AD, Chen Y, Tian M, et al. The human mu opioid receptor: modulation of functional desensitization by calcium/calmodulin-dependent protein kinase and protein kinase C. J Neurosci. 1995;15(3 Pt 2):2396-406.

15. Benarroch EE. Oxytocin and vasopressin. Neurology. 2013;80(16):1521.

16. Wamelen DJv, Aziz NA, Zhao J, Balesar R, Unmehopa U, Roos RAC, et al. Decreased hypothalamic prohormone convertase expression in Huntington disease patients. Journal of Neuropathology and Experimental Neurology. 2013;72(12):1126-34.

17. Viale A, Ortola C, Hervieu G, Furuta M, Barbero P, Steiner DF, et al. Cellular localization and role of prohormone convertases in the processing of pro-melanin concentrating hormone in mammals. J Biol Chem. 1999;274(10):6536-45.

18. Breton C, Schorpp M, Nahon J-L. Isolation and characterization of the human melanin-concentrating hormone gene and a variant gene. Molecular Brain Research. 1993;18(4):297-310.

19. Tanga N, Kuboyama K, Kishimoto A, Kiyonari H, Shiraishi A, Suzuki R, et al. The PTN-PTPRZ signal activates the AFAP1L2-dependent PI3K-AKT pathway for oligodendrocyte differentiation: Targeted inactivation of PTPRZ activity in mice. Glia. 2019;67(5):967-84.

20. Wills ZP, Mandel-Brehm C, Mardinly AR, McCord AE, Giger RJ, Greenberg ME. The nogo receptor family restricts synapse number in the developing hippocampus. Neuron. 2012;73(3):466-81.

21. Ngan ESW, Sit FYL, Lee K, Miao X, Yuan Z, Wang W, et al. Implications of endocrine gland-derived vascular endothelial growth factor/prokineticin-1 signaling in human neuroblastoma progression. Clin Cancer Res. 2007;13(3):868-75.

22. Gloor S, Odink K, Guenther J, Nick H, Monard D. A glia-derived neurite promoting factor with protease inhibitory activity belongs to the protease nexins. Cell. 1986;47(5):687-93.

23. Adorjan I, Tyler T, Bhaduri A, Demharter S, Finszter CK, Bako M, et al. Neuroserpin expression during human brain development and in adult brain revealed by immunohistochemistry and single cell RNA sequencing. Journal of anatomy. 2019;235(3):543-54.

24. Wang JY, Zhang W, Roehrl VB, Roehrl MW, Roehrl MH. An autoantigen atlas from human lung HFL1 cells offers clues to neurological and diverse autoimmune manifestations of COVID-19. Front Immunol. 2022;13.
